# Screening and Comparative Efficacy of Indigenous Entomopathogenic Fungi from Forest Ecosystems Against *Culex pipiens* Biotype *molestus* Larvae: Identification of High-Virulence Isolates for Biocontrol Applications

**DOI:** 10.3390/insects17040361

**Published:** 2026-03-25

**Authors:** Spyridon Mantzoukas, Chrysanthi Zarmakoupi, Ioannis Lagogiannis, Panagiotis A. Eliopoulos

**Affiliations:** 1 Institute of Mediterranean Forest Ecosystems, ELGO-Demeter, 11528 Athina, Greece; 2 Department of Agriculture, University of Ioannina, Arta Campus, 47100 Arta, Greece; chris.zarm@hotmail.com; 3 Plant Protection Division of Patras, ELGO-Demeter, 26442 Patras, Greece; ilagogiannis@elgo.gr; 4 Laboratory of Plant Health Management, Department of Agrotechnology, University of Thessaly, 41500 Larissa, Greece; eliopoulos@uth.gr

**Keywords:** *Culex pipiens*, entomopathogenic fungi, biological control, *Beauveria bassiana*, *Metarhizium anisopliae*, virulence screening, integrated vector management

## Abstract

The management of *Culex pipiens* (Diptera: Culicidae), key vectors of arboviruses like West Nile virus, necessitates sustainable alternatives to chemical insecticides. This study screened indigenous entomopathogenic fungi (EPF) from forest soils in Achaia, Greece, for their larvicidal efficacy against *Cx. pipiens* biotype *molestus*. Fifteen fungal isolates were obtained via insect baiting and identified as *Beauveria* and *Metarhizium* species. A comprehensive bioassay at 1 × 10^8^ conidia mL^−1^ revealed significant variation in pathogenicity after 72 h. Two isolates, *Beauveria bassiana* (BB) (Hypocreales: Cordycipitaceae) and *Metarhizium anisopliae* (K3(1)) (Hypocreales: Clavicipitaceae), exhibited the highest virulence among the tested isolates, each causing 60% mortality with a rapid median lethal time (LT_50_) of ~18.5 h. Survival analysis, Cox modeling, and non-linear kinetic modeling (Gompertz/Richards) classified three distinct virulence clusters: high/rapid, moderate/consistent, and low/delayed. A pathogenicity network analysis and a composite virulence index further validated BB and K3(1) as the most effective candidates. These results demonstrate the high isolate specificity of fungal efficacy and underscore the importance of screening local fungal diversity. The identified high-virulence isolates represent promising, environmentally sound candidates for the development of targeted biopesticides. Future research should focus on formulation for aquatic environments and integration into resistance-resilient integrated vector management programs.

## Simple Summary

Mosquitoes are key vectors for serious diseases, such as West Nile virus. To reduce reliance on chemical sprays, which can harm the environment and lead to resistant mosquitoes, scientists are looking for natural enemies of mosquito larvae. In this study, we screened for beneficial, insect-killing fungi in forest soils from Greece. We isolated several types of these fungi and subsequently tested their efficacy in the laboratory against the larvae of the common house mosquito, *Culex pipiens*. We discovered that two fungal strains were highly effective, causing 60% mortality within three days. Through advanced analysis, we were able to precisely rank how fast and how deadly each fungus was. The best-performing isolates show potential for further development as part of a natural, environmentally friendly mosquito larvicide, though additional research is needed before such products can be commercialized. This research helps pave the way for new, sustainable tools to control disease-carrying mosquitoes.

## 1. Introduction

Mosquitoes (Diptera: Culicidae) play a critical role as dipteran vectors and are biological and mechanical transmitters of numerous parasites and pathogens causing several communicable human and animal diseases globally [1]. They spread enzootic and potentially epizootic diseases, such as malaria, dengue fever, and filariasis [2]. The World Health Organization (WHO) has introduced the Global Vector Control Response (GVCR, 2017–2030) to support the adoption of sustainable vector control policies, with recent updates highlighting the ongoing challenges in vector management [3,4].

Among culicids, the *Culex pipiens* (Diptera: Culicidae) complex is a critical vector of pathogens including West Nile virus, Usutu virus, and avian malaria protozoans [5,6,7]. The complex can also harbor *Wolbachia* endosymbionts, which influence reproductive success via cytoplasmic incompatibility and may affect vector competence, offering novel avenues for biocontrol [8,9]. The widespread geographical distribution of *Cx. pipiens* biotype *molestus* in tropical and subtropical regions underpins its significant socio-economic impact, with recent comprehensive reviews documenting its expansion across Europe and beyond [10,11].

Conventional vector control has heavily relied on chemical insecticides. Despite their effectiveness, these compounds pose considerable health, environmental, and climatic hazards [12]. A major challenge is the widespread development of insecticide resistance in target populations, compounded by the unsustainable nature of many interventions [13]. Chemical larvicides targeting mosquito breeding sites are particularly problematic; they drive resistance in the targeted species and exert long-term secondary effects on non-target aquatic organisms [14].

Entomopathogenic fungi (EPF) offer several advantages in this context: they are naturally occurring in the environment, have narrow host ranges that typically spare non-target organisms including beneficial insects and vertebrates, can be produced on inexpensive agricultural substrates, and have the potential for horizontal transmission and recycling in the environment, which may provide sustained control with fewer applications [15]. Additionally, their complex mode of action involving multiple enzymes and toxins reduces the likelihood of resistance development compared to chemical insecticides with single target sites [16].

Entomopathogenic fungi (EPF) represent a prominent group among biological control agents for insect pests [15]. Their application against mosquitoes has shown promise, with species such as *Beauveria bassiana* (Hypocreales: Cordycipitaceae) and *Metarhizium anisopliae* (Hypocreales: Clavicipitaceae) identified as effective against *Cx. pipiens* larvae [17,18]. The pathogenesis of these fungi involves conidial attachment, cuticle penetration via enzymatic activity, proliferation within the hemocoel, and host death, a process that can be modulated by the host’s immune response [19,20]. A critical aspect of EPF efficacy is their isolate-dependent virulence, which is influenced by ecological niche and habitat [17]. Therefore, systematic screening of indigenous isolates from diverse environmental sources represents an important approach to discover novel, high-virulence strains suited for regional biocontrol programs, as demonstrated by recent successful isolations of wild fungal strains from various habitats [21,22,23].

Standardized bioassay methods, including soil baiting with insect hosts and static immersion tests with conidial suspensions, are crucial for reliable efficacy comparisons [24,25]. Recent studies employing advanced statistical and modeling approaches have deepened our understanding of EPF pathogenicity. Analyses such as two-way ANOVA, Kaplan–Meier survival curves, Cox proportional hazards models, and non-linear kinetic modeling (Gompertz and Richards models) provide robust frameworks for comparing isolate virulence, speed of kill (LT_50_), and infection dynamics [26,27]. These methods reveal that pathogenicity is a multidimensional trait, with isolates clustering into distinct groups based on virulence and temporal action [28,29]. High-virulence isolates often exhibit rapid, exponential mortality kinetics, while low-virulence isolates show delayed, inefficient infection progression [30,31].

EPF infection induces profound physiological stress, notably disrupting the host’s carbohydrate metabolism. Infection alters key enzyme activities, depleting energy reserves to fuel fungal growth [32,33]. The fungal production of cuticle-degrading enzymes and toxic secondary metabolites underpins the complex host–pathogen interaction [34,35]. The ultimate success of fungal biocontrol depends on a complex interplay of fungal virulence determinants, host physiological state, and environmental conditions [36,37].

The commercial development of EPF-based biopesticides is advancing, with numerous products available globally [38,39]. Yet the discovery and characterization of high-performing indigenous isolates remains a valuable pursuit for several reasons: (i) indigenous strains may demonstrate superior adaptation to local environmental conditions, potentially enhancing field efficacy; (ii) locally sourced isolates may face simplified registration pathways in some countries, reducing time to market; (iii) maintaining a diverse pipeline of candidate strains from different geographic origins and habitats provides genetic resources that could prove valuable if resistance develops to widely used commercial strains; and (iv) region-specific products can support local economies and reduce dependence on imported biocontrol agents [40,41].

This study aimed to isolate indigenous entomopathogenic fungi from soil samples in Achaia, Greece, and to evaluate their comparative larvicidal efficacy against *Cx. pipiens* biotype *molestus* larvae. Using a multidimensional analytical framework—including pathogenicity screening, time-course mortality analysis, survival modeling, and non-linear kinetic modeling—we sought to identify and characterize high-virulence candidates. The integration of network analysis and composite pathogenicity indices provided a robust quantitative evaluation of isolate performance, contributing viable candidates for future biocontrol formulations and application strategies.

## 2. Materials and Methods

### 2.1. Soil Sampling and Collection of Fungal Isolates

Soil samples were collected to isolate indigenous entomopathogenic fungal (EPF) strains. Samples were taken randomly from various field sites across the prefecture of Achaia, Greece. The prefecture of Achaia in western Greece was selected as the sampling area due to its remarkable ecological diversity and limited previous exploration for entomopathogenic fungi. Additionally, the region’s forest ecosystems have remained undisturbed, increasing the likelihood of recovering indigenous strains with unique virulence characteristics adapted to local conditions. Surface litter was removed at each site, and a sterile soil core borer was used to extract soil from a depth of 0–10 cm. Samples were placed in labeled, sterile plastic bags, stored at 4 °C during transport, and processed within 48 h. In the laboratory, soil was spread on clean cardboard for 24 h at room temperature to reduce excess humidity. This step was critical to minimize the activity of entomopathogenic nematodes, which could interfere with fungal isolation by preying on the insect baits.

Insect Baiting: Following drying, soil was sieved (2 mm mesh) to remove debris and placed in sterile 90 mm Petri dishes. A diverse panel of bait insect species was employed to maximize the recovery of fungal isolates with different host preferences and ecological strategies. The species selected—*Tribolium confusum* (Coleoptera: Tenebrionidae), Sitophilus zeamais (Coleoptera: Curculionidae), *Rhyzopertha dominica* (Coleoptera: Bostrichidae), and *Galleria mellonella* (Lepidoptera: Pyralidae)—represent different orders, feeding guilds, and ecological niches. The inclusion of multiple coleopteran species, which are common soil-dwelling insects in many habitats, increases the chances of detecting fungi that may have co-evolved with these hosts [26]. This multi-bait approach has been recommended in standard isolation protocols to *G. mellonella* and is particularly well-established as a universal bait for entomopathogenic fungi due to its high susceptibility and ability to recover diverse fungal species [27]. This multi-bait approach has been recommended in standard isolation protocols to overcome the selectivity of single bait species and obtain a more comprehensive representation of the indigenous EPF community [28]. Ten larvae from the above species were added to each dish as bait insects. Each soil sample was assayed in triplicate (30 insects total per sample). Inoculated dishes were sealed with Parafilm^®^ and incubated in darkness at 25 ± 1 °C for 14 days. Cadavers were examined daily, and insects showing signs of mycosis (discoloration, fungal outgrowth) were removed for fungal isolation.

### 2.2. Isolation and Purification of Fungal Isolates

Dead, mycosed insects were surface-sterilized by immersion in 1% sodium hypochlorite for 2 s, followed by two rinses in sterile distilled water. They were then placed on Sabouraud Dextrose Agar (SDA) plates supplemented with 0.1% chloramphenicol to suppress bacterial growth. Alternatively, conidia from sporulating cadavers were directly streaked onto SDA plates. Plates were incubated at 25 ± 1 °C in darkness. Fungal colonies emerging from cadavers were sub-cultured onto fresh SDA plates to obtain pure isolates. Purification was achieved through successive single-spore isolation or hyphal tipping. Isolates were preliminarily identified based on macro- and micromorphological characteristics (colony morphology, conidiophore structure, conidial shape and size) using taxonomic keys.

### 2.3. Fungal Isolates and Culture Maintenance

Fifteen fungal isolates obtained through the above method were used in this study. All isolates were maintained on SDA slants at 4 °C for short-term storage and in 15% glycerol at −80 °C for long-term preservation. For bioassay preparation, each isolate was cultured on 90 mm Ø Petri dishes containing Sabouraud Dextrose Agar and incubated in complete darkness at 25 ± 1 °C for 15 days to promote abundant conidiation. Plates were sealed with Parafilm^®^ to prevent contamination and desiccation.

### 2.4. Conidial Suspension Preparation

Fresh conidia were harvested from 15-day-old cultures by flooding plates with 10 mL of sterile 0.05% aqueous Tergitol^®^ NP9 (nonylphenol ethoxylate) solution and gently scraping the surface with a sterile loop. The suspension was filtered through four layers of sterile muslin cloth into a 500 mL glass beaker to remove hyphal fragments. The concentration of conidia was determined using a Neubauer hemocytometer (Weber Scientific, Hamilton, NJ, USA). The stock suspension was adjusted with sterile 0.05% Tergitol^®^ solution to a final concentration of 1 × 10^8^ conidia mL^−1^ for all isolates, a standard dose widely used in comparable bioassays. Conidial viability was confirmed to be >97% for all isolates by plating appropriate dilutions on SDA and assessing germination (>100 conidia counted) after 18–24 h incubation at 25 °C. Suspensions were used within 2 h of preparation.

### 2.5. Mosquito Rearing and Larval Source

*Cx. pipiens* biotype *molestus* larvae were obtained from a laboratory colony maintained at Entomology Lab of Institute Mediterranean Forest Ecosystems in Athens. The colony was reared under standard insectary conditions: 27 ± 2 °C, 75 ± 10% relative humidity, and a 12:12 h light:dark photoperiod. Larvae were fed a standardized diet of finely ground rodent chow and brewer’s yeast (3:1 ratio). For bioassays, healthy, early 3rd instar larvae of uniform size were selected.

### 2.6. Bioassay Procedure

Larvicidal activity was evaluated using a static immersion bioassay. For each fungal isolate and the control (0.05% Tergitol^®^ solution only), five replicates were established. Each replicate consisted of a 50 mL plastic cup containing 20 mL of dechlorinated tap water and 10 larvae (with five replicates per treatment, this resulted in n = 50 larvae per treatment). A total of 2 mL of the appropriate conidial suspension was added to each cup, achieving the final test concentration of 1 × 10^8^ conidia mL^−1^. Larvae were provided with a minimal amount of food (0.5 mg) 2 h prior to treatment to standardize nutritional status without fouling the water.

Mortality was recorded at 12, 24, 48, and 72 h post-treatment (hpt). Larvae were considered dead if they showed no movement upon gentle prodding with a fine brush. Dead larvae were removed at each observation to prevent saprophytic growth.

### 2.7. Data and Statistical Analysis

Cumulative percentage mortality was calculated for each treatment at each time point. Statistical analyses were performed using R software (v4.3.0, R Core Team, 2023). The effects of Treatment (fungal isolate), Time, and their interaction on larval mortality were analyzed using a two-way repeated measure Analysis of Variance (ANOVA). Where significant main effects were found, Tukey’s Honestly Significant Difference (HSD) post hoc test was used for multiple comparisons between treatment means at 72 hpt. Survival data were analyzed using Kaplan–Meier survival curves, and differences among treatments were compared with the log-rank test. Hazard ratios (HR) with 95% confidence intervals were estimated using a Cox proportional hazards model. The median lethal time (LT_50_) and associated 95% confidence intervals (CI) were estimated by fitting time-mortality data to non-linear models (Gompertz and Richards) using the drc package in R. Time-course mortality data for each strain were fitted to these non-linear models to characterize and compare infection kinetics (growth rate, inflection point). The standardized effect size for differences between each treatment and the control was calculated using Cohen’s *d*. All tests were considered significant at α = 0.05.

### 2.8. Pathogenicity Network Analysis

To integrate multiple parameters of EPF virulence into a unified comparative framework, a pathogenicity network analysis was performed. For each fungal isolate, nodes represented individual strains, while edges reflected similarity based on a combination of final cumulative mortality (72 hpt), median lethal time (LT_50_), and temporal mortality patterns (early vs. delayed action). Network construction was based on standardized values of these parameters, allowing for visualization of isolate clustering according to pathogenicity profiles. The network analysis is a visualization tool to identify groups of isolates sharing similar virulence, rather than as a statistical testing method.

### 2.9. Calculation of the Pathogenicity Index

Pathogenicity Index (PI) was calculated for each EPF isolate by integrating three key parameters: final cumulative mortality (%), median lethal time (LT_50_), and onset mortality. Final cumulative mortality was expressed as the percentage of dead insects in each treatment (n = 50) at the end of the experimental period with control mortality corrected using Abbott’s formula. The median lethal time (LT_50_), defined as the time required for the isolate to kill 50% of the test insect population, was calculated by fitting daily mortality data to a Kaplan–Meier survival model. The onset mortality was quantified as the time to the observation of the first confirmed mortality. These three parameters were integrated into a Pathogenicity Index (PI). Prior to integration, raw values for each parameter were standardized to a common scale (0–1) using min–max normalization to account for their different units of measurement. The standardized scores were then combined using a weighted linear composite score, where the weights were assigned to reflect the relative applied importance of each virulence trait in a biocontrol context.

The index was calculated using the following formula:

PI = (Final Mortality × 0.4) + ((100 − LT_50_) × 1.2) + (Onset Score × 0.3)

The onset score was assigned based on the timing of initial significant mortality. The weighting scheme prioritizes a rapid speed of kill (LT_50_), assigning it the greatest influence (weight = 1.2), followed by the ultimate lethal capacity (final mortality, weight = 0.4) and initial infection aggressiveness (onset, weight = 0.3). A higher PI value indicates superior overall pathogenicity. Isolates were subsequently ranked based on their PI scores for comparative analysis. Higher PI values indicate greater overall EPF virulence.

## 3. Results

### 3.1. Isolation of Indigenous Entomopathogenic Fungi

Fungal isolation via the baiting method yielded multiple entomopathogenic fungal (EPF) isolates from soil samples collected across Achaia, Greece. Based on macro- and micromorphological characteristics, the isolates were preliminarily identified as belonging to the genera *Beauveria* and *Metarhizium*. A total of fifteen isolates were selected for pathogenicity screening against *Cx. pipiens* larvae.

### 3.2. Pathogenicity Screening and Comparative Larvicidal Efficacy

A comprehensive bioassay screening all fifteen fungal isolates at a standard concentration of 1 × 10^8^ conidia mL^−1^ conidia revealed significant and highly variable larvicidal activity against 3rd instar *Cx. pipiens* larvae over 72 h.

Statistical analysis confirmed a highly significant effect of fungal treatment on larval mortality (Two-way ANOVA: Treatment effect, F_15,256_ = 26.92, *p* < 0.001). Final cumulative mortality at 72 h post-treatment (hpt) delineated a clear hierarchy of virulence among the tested isolates (Table 1). Two indigenous isolates, BB (*B. bassiana*) and K3(1) (*M. anisopliae*), exhibited the highest virulence, each causing 60.0% cumulative mortality—significantly greater than all other treatments (*p* < 0.001). Isolates BB ΑΧΑΙΑ, E26R, K1 (1A), and E73 BM demonstrated moderate virulence (44.0–48.0%), followed by Δ400 and K2 (38.0–42.0%). Isolates ISARIA, K1 (2), and MET 2 caused 26.0–30.0% mortality, while K1 (8), Δ32B B, K4 (3), and E4R BΔ were the least effective (16.0–22.0%), statistically indistinguishable from the control (0%).

### 3.3. Time-Course Mortality Dynamics and Survival Analysis

Mortality progressed significantly over time (Two-way ANOVA: Time effect, F_3,256_ = 40.29, *p* < 0.001). A significant Treatment × Time interaction (F_45,256_ = 1.76, *p* = 0.004) indicated distinct temporal mortality patterns among isolates.

The high-virulence isolates BB and K3 (1) exhibited rapid onset of action, causing significant mortality (22% and 26%, respectively) within the first 12 h. Their mortality progressed exponentially, reaching near-maximum levels by 48 hpt. In contrast, isolates like BB ΑΧΑΙΑ and E26R showed a steadier, more linear increase in mortality over the 72 h period. Low-virulence isolates (Δ32B B, E4R BΔ) displayed markedly delayed and minimal killing activity.

**Figure 1:**
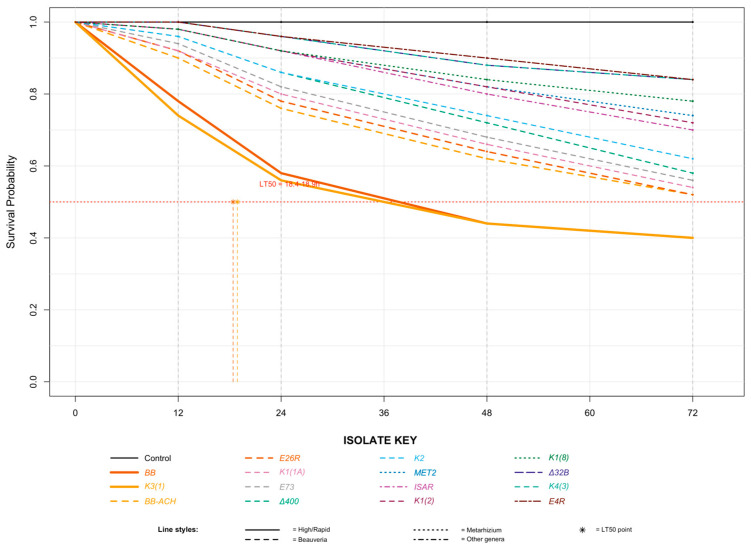
Kaplan–Meier survival curves for *Cx. pipiens* larvae exposed to representative entomopathogenic fungal isolates (1 × 10^8^ conidia mL^−1^).

Survival analysis corroborated these findings. Kaplan–Meier curves differed significantly among treatments (Log-rank test: χ^2^ = 315.6, df = 15, *p* < 0.001) (Figure 1). High-virulence isolates (BB, K3(1)) caused rapid mortality with median survival times of 38.4 h. Moderate-virulence isolates (E26R, BB ΑΧΑΙΑ) showed intermediate survival patterns, while low-virulence isolates (E4R BΔ) exhibited survival curves approaching the control. Differences among curves were highly significant (Log-rank test: χ2 = 315.6, df = 15, *p* < 0.001). BB and K3 (1) drastically reduced larval survival compared to the control and all other isolates (Table 2).

A Cox proportional hazards model quantified the mortality risk. Larvae exposed to BB had an 8.45-fold higher instantaneous risk of death (HR = 8.45, 95% CI: 5.12–13.94, *p* < 0.001) compared to controls, while those exposed to K3 (1) had a 7.82-fold higher risk (HR = 7.82, 95% CI: 4.74–12.91, *p* < 0.001). The control group (0.05% Tergitol^®^) showed 100% survival throughout the 72 h observation period.

### 3.4. Pathogenicity Network Analysis and Virulence Clustering

**Figure 2:**
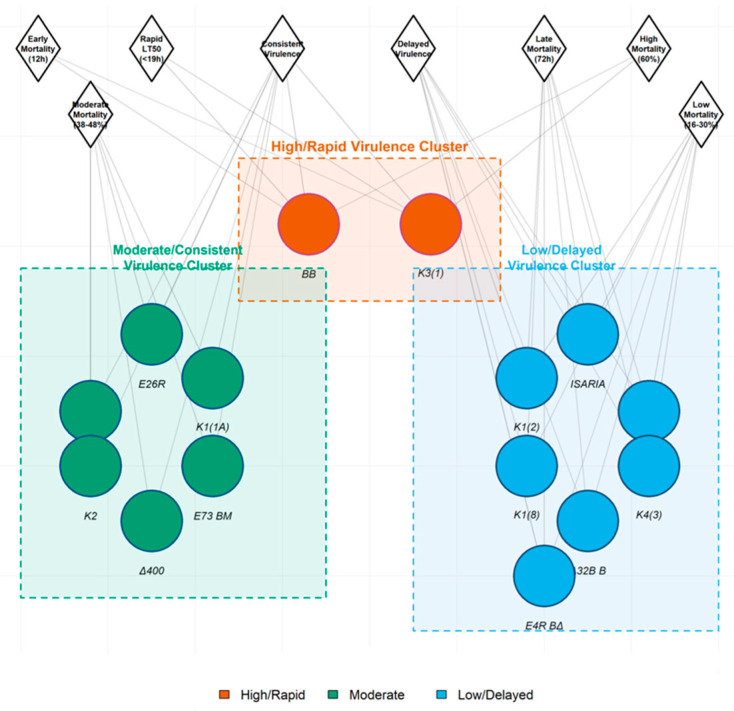
Pathogenicity network analysis of entomopathogenic fungal isolates against *Cx. pipiens* larvae. Nodes represent individual fungal isolates (colored shapes) and virulence traits (gray diamonds). Edges connect isolates to their associated traits based on final cumulative mortality (72 h), median lethal time (LT_50_), and temporal mortality patterns (onset and progression). Three distinct virulence clusters are evident: Cluster 1 (High/Rapid Virulence)—isolates BB and K3(1) showing strong connections to “Early Mortality (12 h)” and “Rapid Virulence” traits; Cluster 2 (Moderate/Consistent Virulence)—isolates BB ΑΧΑΙΑ, E26R, K1(1A), E73 BM, Δ400, and K2 linked to “Consistent Virulence” and intermediate mortality timing; Cluster 3 (Low/Delayed Virulence)—isolates MET 2, K1(8), Δ32B B, K4(3), and E4R BΔ associated with “Delayed Virulence” and “Late Mortality (72h)”. Network analysis integrates multidimensional pathogenicity data to visualize isolate groupings based on shared virulence profiles.

Based on the pathogenicity network analysis described in Section 2.8, the complex relationships between isolates were visualized (Figure 2). This network integrated several factors such as final mortality, speed of kill (LT_50_), and temporal mortality patterns (early vs. late action).

The analysis revealed three primary virulence clusters: High & Rapid Virulence Cluster: Centered on isolates BB and K3(1), characterized by strong connections to “Early Mortality (12 h)” and “Rapid Virulence” nodes. This cluster represents isolates with immediate and potent pathogenic action. Moderate & Consistent Virulence Cluster: Encompassing isolates such as BB ΑΧΑΙΑ, E26R, K1 (1A), E73 BM, Δ400, and K2. These isolates showed strong linkages to the “Consistent Virulence” node, indicating steady mortality progression over time, and moderate connections to both early and late mortality. Low & Delayed Virulence Cluster: Containing isolates MET 2, K1 (8), Δ32B B, K4 (3), and E4R BΔ. This cluster was strongly associated with “Delayed Virulence” and “Late Mortality (72 h)” nodes, reflecting their slow, inefficient infection processes.

This network visualization confirms that pathogenicity is not a linear spectrum but a multidimensional trait, effectively grouping isolates by their shared kinetic profiles and ultimate efficacy.

### 3.5. Modeling Infection Kinetics: Gompertz vs. Richards Model Flexibility

To quantitatively describe and compare the diverse infection kinetics implied by the network clusters, time-mortality data were fitted to two non-linear growth models: the Gompertz and the Richards models. Model comparison via Akaike Information Criterion (AIC) and analysis of residuals revealed the differential flexibility of these models in capturing strain-specific mortality patterns.

The Gompertz model, characterized by a fixed inflection point, provided an excellent fit (*R*^2^ > 0.95) for isolates in the “High & Rapid Virulence” cluster (BB and K3 (1)), which exhibit classic exponential mortality. However, for isolates in the “Low & Delayed Virulence” cluster (Δ32B B, E4R BΔ), the Gompertz model showed systematic bias.

The Richards Model Flexibility Analysis demonstrated this model’s superior adaptability. The shape parameter (*ν*) in the Richards equation, which determines the symmetry and position of the inflection point, varied significantly across isolates (F(14.70) = 15.4, *p* < 0.001. Isolates in the rapid-killing cluster had *ν* values close to 1 (indicating a symmetric, Gompertz-like curve). In contrast, isolates in the delayed-action cluster exhibited *ν* values significantly greater than 1 (Δ32B B, *ν* = 3.12), confirming a right-skewed curve with a delayed inflection point, which the Richards model captured precisely. This quantitative analysis aligns with the clusters identified in the network, confirming that mortality progression is not uniform and model selection must match the isolate’s pathogenic behavior.

### 3.6. Lethal Time Estimation and Pathogenicity Indices

Leveraging the best-fit models (Gompertz for rapid isolates, Richards for delayed-action isolates), median lethal time (LT_50_) values were estimated. The most virulent isolates, BB and K3 (1), had the lowest LT_50_ values of 18.4 h and 18.9 h, respectively (Table 3). Pathogenicity Index ranked BB and K3 (1) highest (PI = 92.5), confirming their superior and rapid larvicidal activity, among tested isolates.

### 3.7. Statistical Effect Size and Power

The standardized effect size (Cohen’s *d*) for treatment differences relative to the control was “Very Large” for the top performers (BB and K3 (1), *d*= 2.85) and ranged to “Medium” for the low-virulence isolates (E4RBΔ, *d*= 0.76). A post hoc power analysis confirmed the experimental design provided sufficient power (>0.99) to detect the significant effects of the high-virulence isolates.

## 4. Discussion

The development of entomopathogenic fungi (EPF) as biocontrol agents has progressed significantly, with numerous wild strains being isolated and evaluated for their insecticidal potential against a range of pest species [22,41], and commercial products now available for managing various insect pests worldwide [38,39]. Our findings contribute to this growing field, demonstrating significant and variable larvicidal efficacy of several fungal isolates against *Cx. pipiens*. The observed virulence spectrum, with strains BB and K3(1) achieving 60% mortality and others like Δ32B B showing only 16%, underscores the critical principle that pathogenicity is highly isolate-dependent and strain-specific. This supports the growing evidence that ecological niche and habitat selection are primary drivers of fungal virulence traits [42,43]. Consequently, the screening pipeline for potential biocontrol isolates should not be confined to those originating from the target host but must be expanded to encompass environmentally diverse sources to discover novel, high-virulence strains [23,44].

In summary, the screening identified two indigenous fungal isolates, BB (*B. bassiana*) and K3(1) (*Metarhizium* sp.), as highly virulent and rapid-acting candidates for the biological control of *Cx. pipiens* larvae. The integration of network analysis and non-linear kinetic modeling provided a multidimensional and quantitative framework for classifying isolate pathogenicity, distinguishing between rapid exponential kill, consistent progression, and delayed, inefficient pathogenesis. The mortality dynamics observed in our study align with established models of fungal pathogenesis. The positive correlation between mortality and time (or, in dose–response studies, concentration) is well-documented and attributed to increased spore load, enhancing the probability of successful cuticle attachment, germination, and host invasion [45,46,47]. The disparity in efficacy between fungal species and isolates can be primarily attributed to their differential ability to secrete a complex arsenal of cuticle-degrading enzymes and secondary metabolites [48]. This is consistent with previous work showing that *B. bassiana* at high concentrations (1 × 10^8^ spores/mL) can cause high mortality in *Culex quinquefasciatus* (Diptera: Culicidae), with efficacy declining proportionally with concentration and larval age [49]. Similarly, studies on *Aedes aegypti* (Diptera: Culicidae) and *Anopheles* spp. (Diptera: Culicidae) have repeatedly demonstrated that early larval instars suffer significantly higher mortality than later ones when exposed to fungi like *Leptolegnia chapmani* (Saprolegniales: Leptolegniaceae) and *M. anisopliae* [50,51]. The pathogenesis typically involves spore germination on the cuticle, penetration into the hemocoel, proliferation, and eventual death via a combination of mechanical obstruction, nutrient depletion, and toxin-induced organ failure, which may take 3–5 days to culminate, sometimes during the vulnerable pupal stage [52,53].

The virulence levels observed in our study (60% mortality at 1 × 10^8^ mL^−1^ conidia with LT_50_ values of 18.5 h) compare favorably with previous reports on entomopathogenic fungi against *Culex* species. Hamad et al. [19] reported that their most effective local isolate, *B. bassiana* (MARD48), caused 80% mortality in *C. quinquefasciatus* larvae within two days at 1 × 10^7^ mL^−1^ conidia, with an LT_50_ of 3.3 days. In comparison, our BB isolate achieved 60% mortality within 72 h with an LT_50_ of only 18.4 h—demonstrating more rapid action despite similar final mortality rates. Benserradj and Mihoubi [20] reported that *M. anisopliae* isolates from Algeria required 5–7 days to achieve 68–100% mortality in *C. pipiens* larvae, slower than our K3(1) isolate. The rapid killing speed of our isolates (LT_50_ <19 h) is particularly notable, as rapid action is a highly desirable trait for biocontrol agents in disease vector management programs where quick population reduction is critical. Recent bioprospecting in Egypt by Alfiky [33] identified multiple *B. bassiana* and *M. anisopliae* isolates with high virulence against lepidopteran and coleopteran pests, but those isolates were not tested against mosquitoes, highlighting the importance of target-specific screening. The superior performance of our isolates may reflect adaptation to local environmental conditions and the specific target host, supporting the rationale for region-specific bioprospecting efforts.

Beyond physical invasion, EPF infection induces profound physiological stress and metabolic disruption in the host. A key aspect of this disruption is the interference with carbohydrate metabolism, which is critical for insect energy homeostasis. Infection by fungi such as *M. anisopliae* and *B. bassiana* has been shown to alter the activity of key enzymes and invertase in various insect hosts [54,55,56]. The insecticidal efficacy of EPF is highly influenced by several other factors such as the insect’s behavior, population density, age, nutrition, genetic information, environmental conditions, as well as the effect of host physiology and morphology on its sensitivity to biological control agents [57]. Therefore, the differences in insect susceptibility to EPF could not be explained solely as a function of the applied conidial concentration [58].

Median lethal time values indicate that the examined isolates are as effective as any other isolate reported in the literature. Similar median lethal time values have been reported for various insect pests treated with EPF species, including stored product beetles [26,59,60,61,62,63,64]. The development of an epizootic depends on many factors: host properties, pathogen properties, their populations, the environment [58], as well as the interaction between these parameters. The transmission of a fungal pathogen occurs through several processes: conidial production, discharge, dispersal, survival, and germination [65]. However, it should be mentioned that variation, not only in experimentation methods, fungal isolates, and insect strains but also in median lethal time estimation methods, renders the direct comparison of these values challenging.

## 5. Conclusions

This study successfully identified and characterized indigenous entomopathogenic fungi (EPF) from Greek soil samples with high larvicidal activity against *Cx. pipiens*, a key vector of human and animal pathogens. Among the screened isolates, *B. bassiana* (strain BB) and *Metarhizium* sp. (strain K3(1)), causing 60% mortality with a median lethal time (LT_50_) of under 19 h. The application of a multidimensional analytical framework—integrating survival analysis, network clustering, and non-linear kinetic modeling (Gompertz and Richards models)—provided a robust, quantitative classification of isolate virulence, clearly distinguishing rapid, moderate, and delayed pathogenic profiles. The findings underscore a core principle in fungal biocontrol: efficacy is highly isolate-dependent and driven by complex interactions between fungal virulence factors, host physiology, and environmental conditions. The significant virulence observed in these indigenous strains highlights the critical importance of screening locally sourced fungi to discover candidates that are optimally adapted for regional biocontrol programs. These promising, high-virulence isolates represent a viable foundation for developing environmentally sustainable biopesticides. Future research must now focus on formulating these fungi for stability and efficacy in aquatic habitats, assessing their safety for non-target organisms, and exploring their potential for integration into resistance-resilient, integrated vector management strategies.

## Figures and Tables

**Table 1:** Cumulative mortality of *Cx. pipiens* larvae (3rd instar) 72 h post-treatment with various entomopathogenic fungal isolates (Mean ± SE, n = 50). Statistical grouping based on a post hoc test (Tukey’s HSD); isolates sharing the same letter are not significantly different.

Treatment	EPF	Mean Mortality (%) ± SE	Statistical Grouping †	Location	ID	Insect Bait
Co (Control)		0.0 ± 0.0	F			
E4R BΔ	*Aspergillus aflatoxiformans*	16.0 ± 1.0	E	Elos-W. Greece	5TWCN9JS013	*Tribolium confusum*
K4 (3)	*Beauveria* *bassiana*	16.0 ± 0.8	E	Moires-Crete	H9WJ96N9013	*Sitophilus zeamais*
Δ32B B	*Chaetomium longiciliata*	16.0 ± 0.8	E	Dassylio-W. Greece	5TVW5VWE016	*Tribolium confusum*
K1 (8)	*Metarhizium* *anisopliae*	22.0 ± 0.9	E	Kouses-Crete	E7JJ49D9899	*Sitophilus zeamais*
K1 (2)	*Beauveria* *bassiana*	28.0 ± 1.5	D	Kouses-Crete	5TUDVUZA016	*Sitophilus zeamais*
MET 2	*Metarhizium* *anisopliae*	26.0 ± 1.0	D	Glafkos-W. Greece	20140422CS3P3_C02	*Plodia interpunctella*
ISARIA	*Isaria fumosorosea*	30.0 ± 1.0	D	Ag. Stefanos-C. Greece	20170105CS10P1_G01	*Galleria mellonella*
K2	*Beauveria* *bassiana*	38.0 ± 1.2	C	Vagionia-Crete	G9DJ98N9016	*Sitophilus zeamais*
Δ400	*Beauveria* *bassiana*	42.0 ± 1.1	C	Dassylio-W. Greece	5THRJ27U016	*Sitophilus zeamais*
E73 BM	*Beauveria* *bassiana*	44.0 ± 1.3	B	Elos-W. Greece	5TUAMDT0013	*Tribolium confusum*
K1 (1A)	*Beauveria* *bassiana*	46.0 ± 1.3	B	Kouses-Crete	5TTMZK2G013	*Sitophilus zeamais*
BB ΑΧΑΙΑ	*Beauveria* *bassiana*	48.0 ± 1.1	B	Glafkos-W. Greece	20170105CS6P2_I02	*Rhyzopertha dominica*
E26R	*Beauveria* *bassiana*	48.0 ± 1.1	B	Elos-W. Greece	5TSGH8UH016	*Sitophilus zeamais*
K3 (1)	*Metarhizium* *anisopliae*	60.0 ± 1.0	A	Galia-Crete	D9JJ45D9301	*Sitopholus zeamais*
BB	*Beauveria* *bassiana*	60.0 ± 1.1	A	Athens-C. Greece	20140422CS6P8_H02	*Galleria mellonella*

† Tukey’s HSD test: Treatments sharing the same letter are not significantly different (*p* > 0.05).

**Table 2:** Survival metrics for *Cx. pipiens* larvae exposed to selected entomopathogenic fungi.

Treatment	Mean Survival Time (h)	Median Survival Time, ST_50_ (h)	95% CI for ST_50_
Co	>72.0	>72.0	(72.0, >72.0)
BB	43.2	38.4	(31.2, 45.6)
K3 (1)	43.2	38.4	(31.2, 55.2)
BB ΑΧΑΙΑ	48.0	52.8	(38.4, 67.2)
E26R	48.0	52.8	(38.4, 67.2)
E4R BΔ	66.0	>72.0	(62.4, >72.0)

**Table 3:** Estimated lethal time parameters and Pathogenicity Index for key fungal isolates.

Strain	LT_50_ (h)	95% CI for LT_50_	Pathogenicity Index *	Best-Fit Model
BB	18.4	(16.8, 20.0)	92.5	Gompertz
K3 (1)	18.9	(17.3, 20.5)	92.5	Gompertz
BB ΑΧΑΙΑ	26.8	(24.5, 29.1)	78.3	Richards
E26R	27.6	(25.2, 30.0)	78.3	Richards
E4R BΔ	86.1	(79.0, 93.2)	26.0	Richards

* PI = (Final Mortality % × 0.4) + ((100 − LT_50_) × 1.2) + (Onset Score × 0.3); higher scores indicate greater virulence.

## Data Availability

The original contributions presented in this study are included in the article. Further inquiries can be directed to the corresponding author.
